# Alterations in White Matter Microstructure in Neurofibromatosis-1

**DOI:** 10.1371/journal.pone.0047854

**Published:** 2012-10-19

**Authors:** Katherine H. Karlsgodt, Tena Rosser, Evan S. Lutkenhoff, Tyrone D. Cannon, Alcino Silva, Carrie E. Bearden

**Affiliations:** 1 Semel Institute for Neuroscience and Behavior, University of California Los Angeles, Los Angeles, California, United States of America; 2 Center for Cognitive Neuroscience, University of California Los Angeles, Los Angeles, California, United States of America; 3 Department of Psychology, University of California Los Angeles, Los Angeles, California, United States of America; 4 Children’s Hospital Los Angeles, Los Angeles, California, United States of America; 5 Department of Neurobiology, University of California Los Angeles, Los Angeles, California, United States of America; International Centre for Genetic Engineering and Biotechnology, Italy

## Abstract

Neurofibromatosis (NF1) represents the most common single gene cause of learning disabilities. NF1 patients have impairments in frontal lobe based cognitive functions such as attention, working memory, and inhibition. Due to its well–characterized genetic etiology, investigations of NF1 may shed light on neural mechanisms underlying such difficulties in the general population or other patient groups. Prior neuroimaging findings indicate global brain volume increases, consistent with neural over-proliferation. However, little is known about alterations in white matter microstructure in NF1. We performed diffusion tensor imaging (DTI) analyses using tract-based spatial statistics (TBSS) in 14 young adult NF1 patients and 12 healthy controls. We also examined brain volumetric measures in the same subjects. Consistent with prior studies, we found significantly increased overall gray and white matter volume in NF1 patients. Relative to healthy controls, NF1 patients showed widespread reductions in white matter integrity across the entire brain as reflected by decreased fractional anisotropy (FA) and significantly increased absolute diffusion (ADC). When radial and axial diffusion were examined we found pronounced differences in radial diffusion in NF1 patients, indicative of either decreased myelination or increased space between axons. Secondary analyses revealed that FA and radial diffusion effects were of greatest magnitude in the frontal lobe. Such alterations of white matter tracts connecting frontal regions could contribute to the observed cognitive deficits. Furthermore, although the cellular basis of these white matter microstructural alterations remains to be determined, our findings of disproportionately increased radial diffusion against a background of increased white matter volume suggest the novel hypothesis that one potential alteration contributing to increased cortical white matter in NF1 may be looser packing of axons, with or without myelination changes. Further, this indicates that axial and radial diffusivity can uniquely contribute as markers of NF1-associated brain pathology in conjunction with the typically investigated measures.

## Introduction

Neurofibromatosis Type 1 (NF1) is caused by a mutation in the neurofibromin gene at locus 17q11.2, and is one of the most common single-gene genetic disorders (prevalence 1∶3000) affecting cognitive function in humans [1]. It is also characterized by multi-system clinical findings, including including café au lait spots, neurofibromas, scoliosis, bone dysplasias, optic pathway gliomas and Lisch nodules [2]. However, the accompanying cognitive deficits lead to significant difficulties in functioning, particularly in the school environment, and in fact are often the most significant cause of lifetime morbidity in this population [3]. The NF1 cognitive profile is characterized by severe impairments in ‘cognitive control’, or the ability to maintain attentional focus and resist distraction, which is generally considered to be a key function of the frontal lobe. These cognitive control deficits are particularly notable in the areas of working memory, cognitive flexibility, and inhibitory control [1], [4], [5], [6].

About 65% of children with NF1 have sustained attentional difficulties and 38–50% meet diagnostic criteria for ADHD, with the vast majority fulfilling criteria for the inattentive subtype [7], [8]. In addition, a substantial proportion of individuals with NF1 demonstrate social deficits similar to those observed in autism spectrum disorders (ASD) [9], [10]. Due to its well-characterized genetic etiology, investigation of the neural mechanisms underlying these deficits in NF1 could shed light on the pathogenesis of attentional dysfunction and social deficits in the broader population.

Neurofibromin, the NF1 gene product, appears early during embryonic development, with high levels of expression in the brain, suggesting that it is important for the orderly differentiation of CNS neurons [11]. Gray and white matter volume increases relative to healthy individuals have been consistently documented, although the origins and significance of these brain changes is unclear [12], [13], [14]. Overall macrocephaly (i.e. brain circumference greater than the 95^th^ percentile) characterizes about half of children with NF1 [15]. Corresponding to increased overall white matter volume, increased corpus callosum area and/or callosal thickening also appears to be characteristic of NF1 [16], [17], [18]. Also, two studies have found increased corpus callosum size to be associated with lower IQ and poorer performance on measures of academic achievement, abstract concept formation, verbal memory and visual-spatial and motor skills [13], [19], indicating that this structural change has functional importance.

NF1 mutations are known to impact myelin, as the gene encoding the oligodendrocyte-myelin glycoprotein (OMgp) is embedded within an intron of the NF1 gene [20]. This protein has been the focus of much interest as a potential mechanism underlying overproliferation of oligodendrocytes, which may explain structural neuroanatomic findings of enlarged white matter. However, while increases in oligodendrocyte progenitors have been demonstrated in the spinal cords of NF1 mutant mice [21], Lee et al. [22] recently showed that NF1 deletion increases neuroglial progenitor/stem cells (NSCs) in the brainstem, but not in the cortex. Consistent with findings that tumors commonly appear in the optic nerve, hypothalamus, and brain stem but rarely in the cortex, they also found no increased proliferation or gliogenesis in cortex. Therefore, the mechanisms underlying the observed white matter changes still require further investigation. However, few studies have examined indices of brain myelination in adult patients with NF1 to determine how these changes are reflected *in vivo*. Diffusion tensor imaging (DTI) is the only currently available non-invasive method for investigating white matter microstructure and connectivity in vivo, based on patterns of water diffusion in neural tissue [23]. The fractional anisotropy (FA; or directional variability) of diffusion is higher in heavily myelinated fiber tracts, and increases with progressive myelination during development [24]. While traditional magnetic resonance imaging (MRI) allows for only a gross overview of white matter, the current study employs diffusion-based methods to examine the integrity of white matter microstructure and inter-regional axonal connectivity.

**Figure 1:**
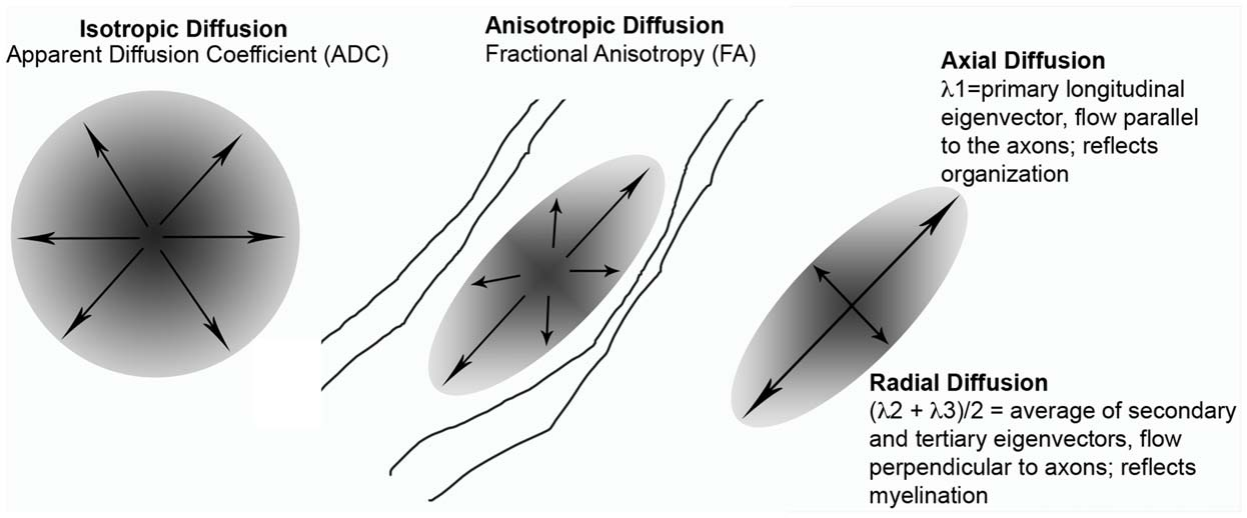
DTI diagram showing radial diffusion, axial diffusion and ADC as related to FA.

To date, only a handful of studies have employed DTI measures in NF1 patients, none of which have taken a whole-brain approach. Previous studies have noted increased apparent diffusion coefficient (ADC) values, indicating more diffuse or less organized cerebral tissue, in both children and adults with NF1 relative to control subjects [25], [26], [27]. More recently, studies have begun exploring the FA properties of white matter using a region of interest (ROI)-based approach. These studies have generally shown decreases in FA, the primary DTI index of white matter microstructural integrity, as well as increases in overall diffusivity [28], [29], [30]. However, these studies have had a limited focus on specific brain regions. A major limitation of an ROI-based approach is that it does not address the possibility that the changes observed may be widespread. In addition, it is not known to what extent the observed increases in overall diffusivity (as indexed by FA and ADC) reflect alterations in radial versus axial diffusion. Axial and radial diffusion measures of the length of the longest and shortest axes of the elliptical area of diffusion (see Figure 1), and are thought to index tract organization or axonal integrity and myelination, respectively [31], [32], [33] particularly in the case of confluent white matter changes [34]. However, it is known that myelination is not the only factor that contributes to radial diffusion [35]. In addition, there may be a role for axonal packing density (amount of space between axons); this measure has been shown to be positively correlated with ADC and radial diffusion, and negatively correlated with FA [36], [37], [38].

Here, we conducted the first study to date to investigate changes in white matter microstructure across the whole brain using voxel-wise methods in NF1. Secondly, we examined volumetric differences in grey and white matter between groups, to determine whether previously observed global volume alterations were also present in our sample. Based on findings from prior mouse and human studies we predicted that individuals with NF1 would have larger overall grey and white matter volumes, concomitant with increases in overall diffusivity and decreased FA. Moreover, since neurofibromin is expressed throughout the brain and not just in the localized regions that were the focus of prior analyses, we hypothesized that a voxel-wise approach would reveal more widespread differences than had been previously reported.

## Methods

### Participants

14 individuals with NF1 and 12 unaffected, demographically matched control subjects participated in the study (see Table 1 for demographics). NF1 participants were recruited via advertisement, and had been previously diagnosed with NF1 by a physician. All NF1 participants fulfilled the diagnostic criteria specified by the National Institutes of Health Consensus Development Conference [39] as confirmed by clinical interview. Healthy controls were recruited from a community sample through advertisements for ongoing research studies at UCLA, and did not have any Axis-I psychiatric disorders, nor any medical conditions that may affect cognitive function, as assessed by the Structured Clinical Interview for DSM-IV [40]. Individuals in NF1 and control groups were excluded for significant substance use in the last six months, history of head injury, mental retardation (IQ less than 70) and/or insufficient fluency in the English language.

**Table 1:** Demographics.

	Patients	Controls	Statistics
**Age** **(yrs ± stdev)**	24±4.93	22.66±4.54	t(24) = .713, p = .483
**Sex (M/F)**	6/8	5/7	X^2^ = .0038, p = .951
**Estimated IQ** **(avg ± stdev)**	96.79±8.68	109.62±9.78	t(20) = 2.189, p = .0046*
**Yrs Education (avg ± stdev)**	14.21±2.12	15.00±2.28	t(24) = -.613, p = .5459
**Race/Ethnicity:**			X^2^ = .1806, p = .671**
**Caucasian**	7	5	
**Hispanic/Latino**	5	1	
**Asian/Pacific Islander**	2	0	
**African** **American**	0	3	
**Other**	0	3	

* IQ data was only available for 8/12 control subjects.

** Race and ethnicity statistics were calculated for Caucasian vs non-Caucasian subjects.

### Ethics Statement

All participants provided written consent for participation, as approved by the institutional review board of the University of California, Los Angeles (UCLA).

**Figure 2:**
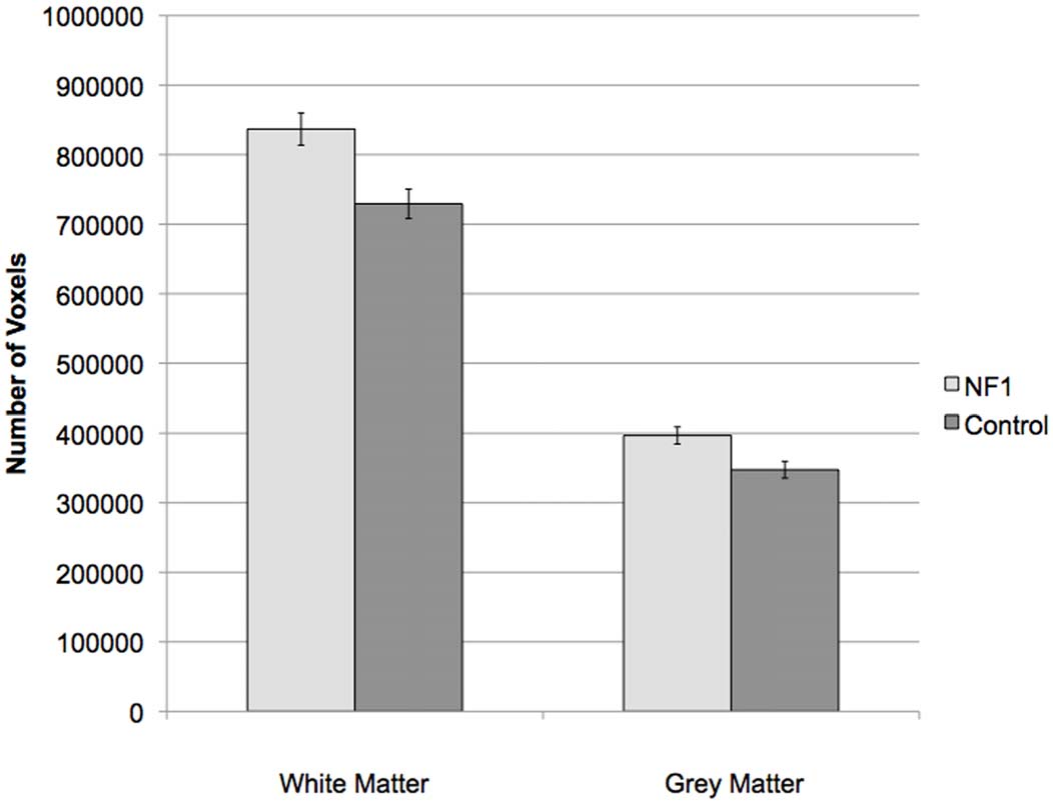
Gray and White Matter volumetric differences between groups.

### Scanning Procedures

Subjects were scanned on a 1.5 T Siemens Sonata scanner (Siemens, Erlagen, Germany) at the Ahmanson-Lovelace Brain Mapping Center at UCLA. Head motion was restricted using foam padding. DTI data were acquired using a 6-direction EPI sequence with 75 contiguous 2 mm AC-PC aligned interleaved slices with no gap (TR = 9.5 s, TE = 77 ms, flip angle = 90 deg, matrix = 128×96, b-value = 1000, FOV 256 mm x 192 mm resulting in 2 mm isotropic voxels). Five repetitions of the sequence were acquired, with a total scan time of 6 minutes 20 seconds. In addition, a high resolution T1-weighted structural image was acquired (1 mm cubic voxels on 160 slices, TR = 1900 ms, TE = 4.38 ms).

**Figure 3:**
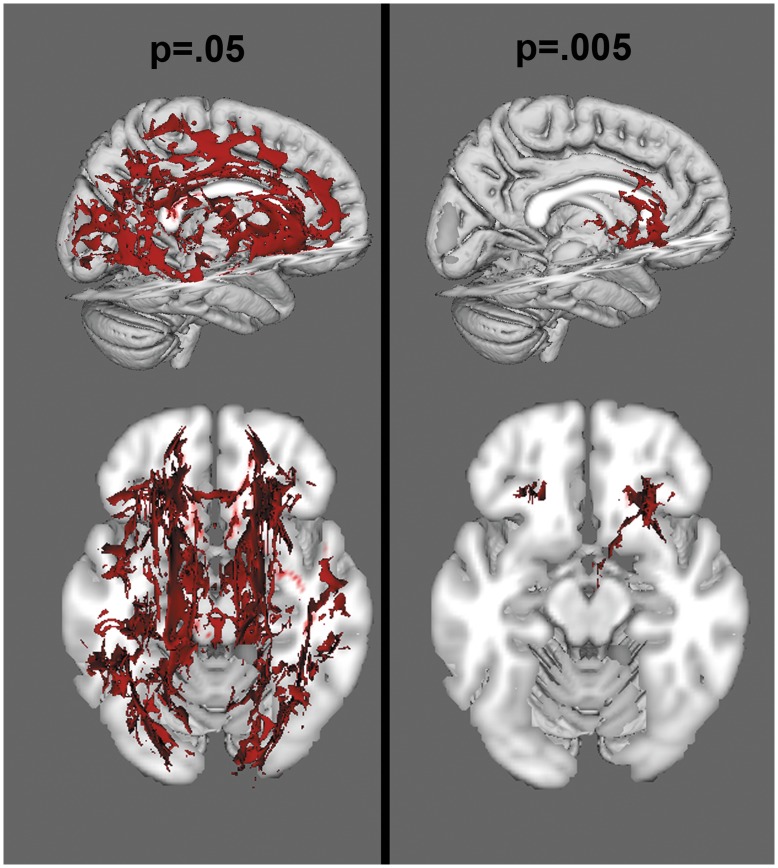
Voxelwise analysis of FA, at a threshold of p<.05 (left) and p<.005 (right). Red colors reflect areas of decreased FA in the NF1 group.

**Figure 4:**
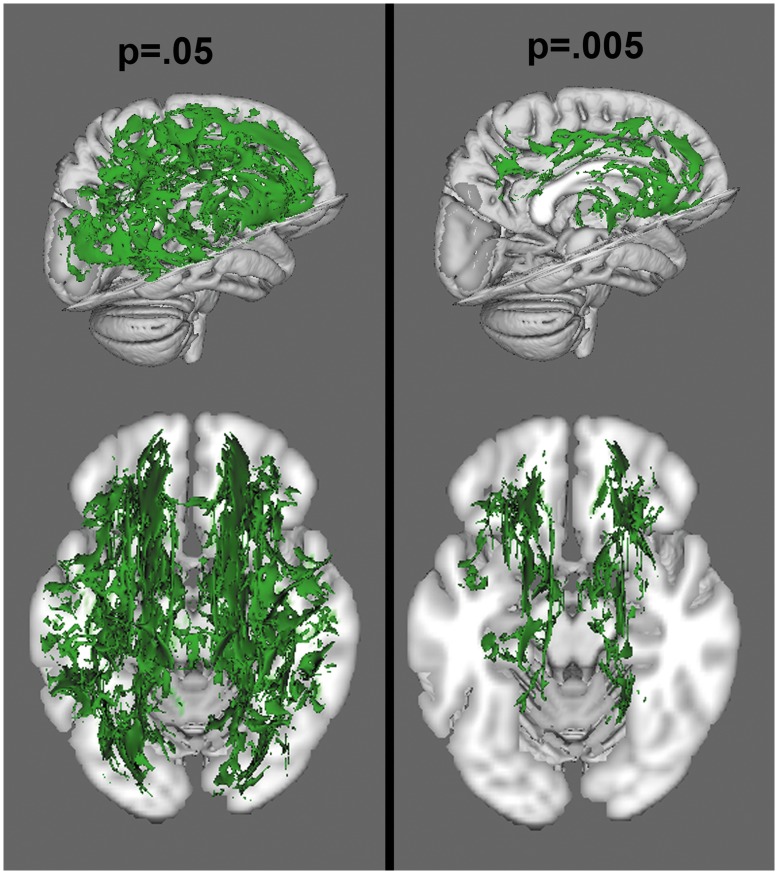
Voxelwise analysis of radial diffusion, at a threshold of p<.05 and p<.005. Green colors reflect increased radial diffusion in the NF1 group.

**Figure 5:**
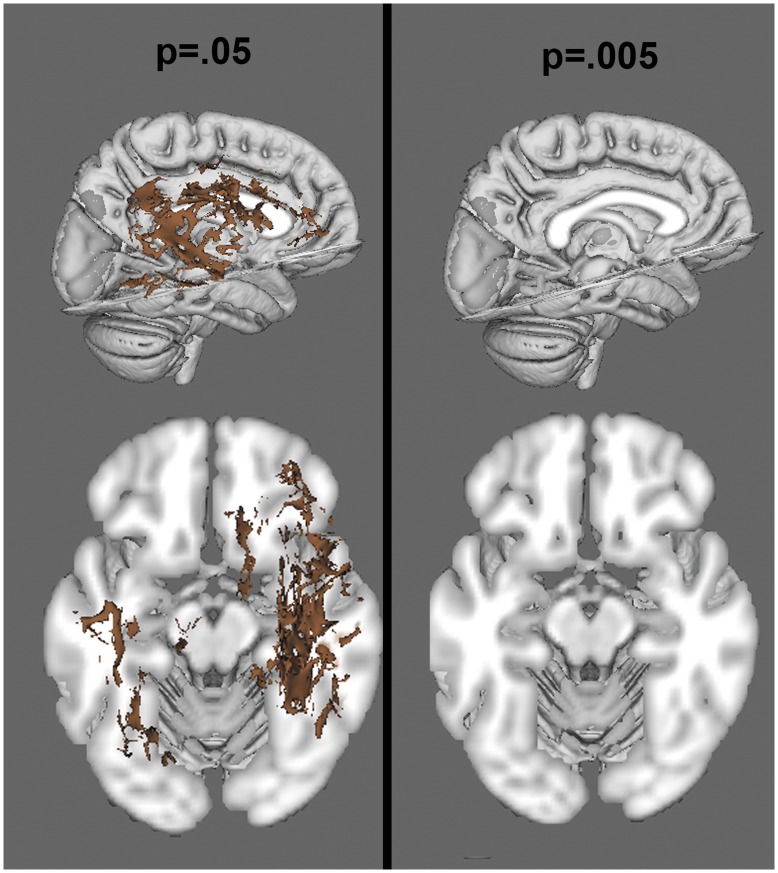
Voxelwise analysis of axial diffusion, at a threshold of p<.05 and p<.005. Brown colors reflect increased axial diffusion in the NF1 group.

**Figure 6:**
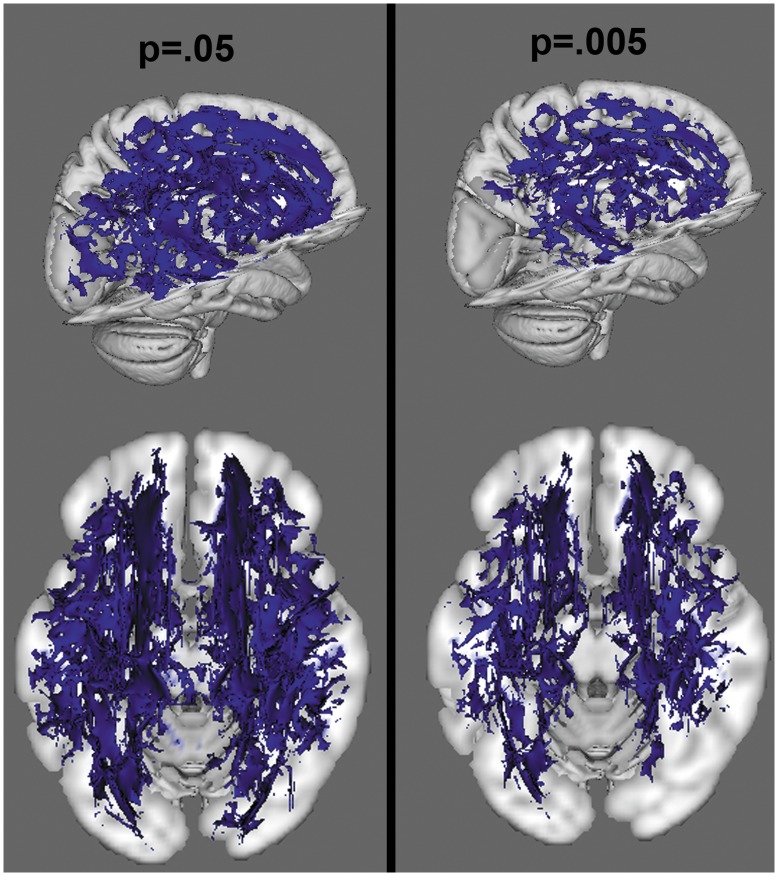
Voxelwise analysis of ADC, at a threshold of p<.05 and p<.005. Blue colors reflect increased ADC in the NF1 group.

### Image Processing

The five image acquisitions for each direction were merged, aligned with McFlirt (FMRIB Software Library; FSL [41]), and averaged to create one file each for the 6 directions and the b0 image. Eddy current correction was done using Flirt (FSL), and images were skull stripped using the Brain Extraction Tool. FA images were calculated using DTIFit (FMRIB’s Diffusion Toolbox), which fits a diffusion tensor model at each voxel, and then were registered to MNI-152 space using a 12-parameter affine registration with a mutual information cost function implemented in Flirt (FSL). A group map was created using Tract-Based Spatial Statistics (TBSS, [42]). An average FA image was created and the tracts were narrowed to generate an FA “skeleton” representing the center of all tracts common to the entire group. The area around the skeleton in each subjects’ aligned FA map was searched and the highest local FA value was assigned to the skeleton. These procedures ensure that each subject’s skeleton is in the group space, yet represents the center of that subject’s own unique fiber tracts. Apparent diffusion coefficient (ADC) was utilized as it is implemented in the FDT toolkit. Radial diffusion was calculated using the value from the primary eigenvector (L1), and axial diffusion was calculated using the average of the secondary and tertiary eigenvectors (L2+L3/2). Each measure was projected on the skeleton and analyzed in TBSS accordingly.

**Table 2:** Differences in Fractional Anisotropy (NF1 Patients vs. Controls).

Region	Hemisphere	Patients	Controls	Statistics
Anterior Thalamic Radiation	L	**.4153 (.0155)**	**.4424 (.0182)**	**t(24) = 4.10, p = .0004; effect size = 1.30**
Anterior Thalamic Radiation	R	**.4171 (.0140)**	**.4480 (.0233)**	**t(24) = 4.16, p = .0003; effect size = 1.6**
Corticospinal Tract	L	.5567 (.0207)	.5654 (.0228)	t(24) = 1.01, p = .321;effect size = .390
Corticospinal Tract	R	.5391 (.0182)	.5433 (.0187)	t(24) = 5.74, p = .572;effect size = .229
Cingulate Bundle	L	**.5253 (.0221)**	**.5639 (.0273)**	**t(24) = 3.99, p = .0005; effect size = 1.24**
Cingulate Bundle	R	.5315 (.0374)	.5555 (.0300)	t(24) = 1.786, p = .087; effect size = .692
Hippocampal Cingulum	L	.3305 (.0195)	.3379 (.0423)	t(24) = .591, p = .2815; effect size = .235
Hippocampal Cingulum	R	.2968 (.0231)	.3093 (.0345)	t(24) = 1.10, p = .282;effect size = .432
Forceps Major	L+R	.7215 (.0351)	.7349 (.0313)	t(24) = 1.023, p = .316; effect size = .402
Forceps Minor	L+R	.5538 (.0186)	.5561 (.0278)	t(24) = 1.838, p = .078; effect size = .691
Inferior fronto-occipital fasciculus	L	.4359 (.0184)	.4592 (.0215)	t(24) = 2.98, p = .0066; effect size = 1.02
Inferior fronto-occipital fasciculus	R	.4329 (.0203)	.4567 (.0204)	t(24) = 2.97, p = .0066; effect size = 1.02
Inferior longitudinal fasciculus	L	.4064 (.0139)	.4178 (.0257)	t(24) = 1.44, p = .164;effect size = .553
Inferior longitudinal fasciculus	R	.4154 (.0175)	.4282 (.0203)	t(24) = 1.72, p = .098;effect size = .652
Superior longitudinal fasciculus	L	.4400 (.0229)	.4550 (.0312)	t(24) = 1.41, p = .172;effect size = .543
Superior longitudinal fasciculus	R	.4538 (.0273)	.4568 (.0208)	t(24) = .312, p = .754;effect size = .125
Uncinate fasciculus	L	.4265 (.0216)	.4308 (.0259)	t(24) = 4.64, p = .647;effect size = .185
Uncinate fasciculus	R	.4072 (.0196)	.4239 (.0260)	t(24) = 1.87, p = .074;effect size = .70

Region of interest based FA analysis.

**Table 3:** Radial and Axial Diffusion and ADC: NF1 Patients vs. Controls**.**

Tract	Measure	Patients	Controls	Statistics
**Left ATR**	**Radial**	**.000614 (2.24×10^−5^)**	**.000579 (2.15×10^−5^)**	**t(24) = 4.097, p = .0004**
	Axial	.001190 (2.67×10^−5^)	.001180 (2.74×10^−5^)	t(24) = .9817, p = .3361
	**ADC**	**.000810 (2.16×10^−5^)**	**.000778 (1.95×10^−5^)**	**t(24) = 3.31, p = .003**
**Right ATR**	**Radial**	**.000603 (2.75×10^−5^)**	**.000563 (2.57×10^−5^)**	**t(24) = 3.79, p = .0009**
	Axial	.001175 (3.09×10^−5^)	.001162 (3.25×10^−5^)	t(24) = .996, p = .3292
	**ADC**	**.000794 (2.74×10^−5^)**	**.000763 (2.22×10^−5^)**	**t(24) = 3.103, p = .0049**
**Left Cingulate**	**Radial**	**.00052 (2.08×10^−5^)**	**.000470 (2.61×10^−5^)**	**t(24) = 5.60, p<.0001**
	Axial	.001260 (4.11×10^−5^)	.001249 (2.87×10^−5^)	t(24) = .803, p = .430
	**ADC**	**.000768 (2.04×10^−5^)**	**.000730 (1.70×10^−5^)**	**t(24) = 5.10, p<.0001**
**Right Cingulate**	**Radial**	**.000515 (3.69×10^−5^)**	**.000480 (3.52×10^−5^)**	**t(24) = .247, p = .021**
	Axial	.001267 (5.47×10^−5^)	.001252 (3.65×10^−5^)	t(24) = .808, p = .427
	**ADC**	**.000766 (2.18×10^−5^)**	**.000737 (2.78×10^−5^)**	**t(24) = 2.93; p = .0073**

Supplementary ROI analysis testing radial diffusion, axial diffusion, and ADC in regions with significant FA findings.

Grey and white matter volumes were assessed using the FSL-VBM tools, in a voxel-based morphometry style analysis [43], [44] carried out with FSL tools [41]. First, structural images were brain-extracted using BET [45]. Next, tissue-type segmentation was carried out using FAST4 [46]. The resulting grey-matter partial volume images were then aligned to MNI152 standard space using the affine registration tool FLIRT [47], [48], followed optionally by nonlinear registration using FNIRT [49], [50], which uses a b-spline representation of the registration warp field [51]. The resulting images were averaged to create a study-specific template, to which the native grey matter images were then non-linearly re-registered. The registered partial volume images were then modulated (to correct for local expansion or contraction) by dividing by the Jacobian of the warp field. The modulated segmented images were then smoothed with an isotropic Gaussian kernel with a sigma of 3 mm. Grey and white matter volumes were determined by counting the number of voxels in each of the segmented (grey and white matter) images. To create a count of voxels in the frontal lobe alone, a mask of the frontal lobe (i.e., brain tissue in front of the central sulcus) was created and warped from MNI space to each individual subjects’ space, and then used as a mask for voxel count extractions.

#### Region of interest definition

White matter regions of interest (ROIs) were defined in the anterior thalamic radiation (ATR), cingulate bundle, hippocampal cingulum, inferior longitudinal fasciculus (ILF), superior longitudinal fasciculus (SLF) and uncinate fasciculus (UF), corticospinal tract, and inferior fronto-occipital fasciculus (IFO) based on the John Hopkins University DTI-based probabilistic tractography atlas [52], [53], [54]. To ensure the validity of the tractography-based ROIs for our TBSS skeleton, all ROIs were overlaid with the skeleton and manually edited. Each subjects’ FA skeleton was masked using each of the ROIs, and the average FA was calculated for each region, as previously described in Karlsgodt et al., 2009 [55].

#### Statistical analysis

Statistics on the ROI data were performed in Stata (v8). Independent samples t-tests were performed on the DTI regions, as well as the structural MRI regions. Voxel-wise statistics on the DTI data were performed in FSL’s randomise tool, which performs a permutation analysis. We performed 5000 permutations using the Threshold Free Cluster Environment (TFCE), a rigorous approach to cluster analysis that avoids the need to define and initial cluster-forming threshold or to perform large amounts of data smoothing. Age and sex (demeaned) were included in the model as confound regressors, with groups modeled separately. Family-wise error rate (FWE) was used to correct for multiple comparisons.

In order to directly test whether the observed FA effects were secondary to radial or axial changes, we performed an exploratory analysis in which we re-ran two additional versions of the FA randomize analysis (again with demeaned age and sex as covariates), once with demeaned radial diffusion and once with demeaned axial diffusion as covariates. Then, the number of remaining significant voxels were compared to the number of original significant voxels in the FA analysis.

## Results

### Structural MRI Results

The structural volume analysis revealed significantly larger total grey matter volume [t(24) = 2.84, p = .009; effect size = .987] and white matter volume [t(24) = 3.38, p = .003; effect size = 1.12] in NF1 patients relative to controls. This finding remained significant when restricted to an ROI of the frontal lobe white matter [t(24) = 3.62, p = .0014; effect size (Cohen’s *d*) = 1.17], however, frontal lobe grey matter was significantly larger in controls [t(24) = 3.64,p = .0013; effect size = 1.174; see Figure 2].

### DTI Results

When corrected for multiple comparisons at p<.05, permutation analysis revealed highly significant reductions in FA in NF1 patients relative to controls, which were spread broadly across the entire brain (Figure 3). Radial diffusion showed a similar pattern of global changes (here, NF1 patients were higher than controls) (Figure 4). However, there were less widespread increases in axial diffusion in the NF1 group (Figure 5). Finally, NF1 patients showed overall increases in ADC (Figure 6) across the brain, at both high and lower thresholds.

Given the widespread nature of the findings at conventional thresholding levels, we took two approaches to ascertain whether these effects were more pronounced in any particular region. First, we performed a secondary voxel-wise analysis at an increased statistical threshold (p = .005) to look for areas that remained significant at suprathreshold levels, indicating more robust differences. We found that the alterations in FA (Figure 3) and radial diffusion (Figure 4) did show regional effects, with only voxels in the frontal lobe surviving the more stringent thresholding. However, increased thresholding for axial diffusion (Figure 5) and ADC maps (Figure 6) did not reveal any suprathreshold patterns: no voxels survived the higher thresholding for axial diffusion, and for ADC the affected regions remained significant throughout the brain. In the exploratory analysis of the relationship between FA and radial and axial diffusion we found that when axial diffusion was entered as a covariate, it did not impact the overall FA findings and 98.23% of the voxels remained the same as in the original FA analysis. However, when radial diffusion was used as the covariate, only 63.32% of voxels remained significant, indicating that radial diffusion may specifically contribute to the FA changes more than axial diffusion.

Our second approach to probing regional variation was to perform a ROI analysis in a set of white major matter association tracts (ROI definition described above) including those that do and do not connect to the frontal lobe. When corrected for multiple comparisons using Bonferroni correction, the significant p-value threshold was set at.005 (.05/10 = .005) (see Table 2). At this threshold the left and right anterior thalamic radiations (ATR), which connect the thalamus and frontal lobes, showed significantly lower FA in NF1 patients, as did the left cingulate bundle. There were trend level effects in the forceps minor, a tract that travels through the genu of the corpus callosum and connects the two hemispheres of the frontal lobe, and the inferior fronto-occipital fasciculus (which projects from the frontal lobe back through the temporal and occipital lobes) bilaterally. Finding significant results in tracts that originate in the frontal lobe confirmed the predominantly frontal nature of the FA alterations (See Figure 2). To probe the basis of these differences, a post-hoc analysis was performed within ROIs showing significant differences in FA; namely, the left and right ATR and the left cingulate bundle (see Table 3), this time assessing radial and axial diffusion as well as ADC. We found that while radial diffusion and ADC were significantly increased in these ROIs, there were no significant differences between groups in axial diffusion. Given the more robust effects in radial diffusion in both the voxelwise and ROI approaches, this convergent evidence supports the hypothesis that the observed FA changes may be driven by alterations in radial diffusion.

## Discussion

This is the first paper to investigate diffusion tensor imaging measures in adults with NF1 across the entire brain using a rigorous registration approach, tract-based spatial statistics (TBSS). First, our data replicate and support previous sMRI findings indicating white matter volumetric enlargement in NF1 patients [12], [13], [14]. However, our study sheds new light on the widespread nature of white matter microstructural alterations in NF1, a pattern that has not been observed in previous studies that focused on a limited selection of white matter tracts. Furthermore, by using two approaches (ROI analysis and a suprathreshold voxel-wise analysis) to probe these widespread effects for regional specificity, we were able to determine that the alterations are more pronounced in the frontal lobe. The finding that alterations in white matter integrity are most pronounced in the frontal lobe in adults with NF1 is highly consistent with the nature of their cognitive deficits. We have previously demonstrated deficits in frontally-mediated working memory processes [1]. Moreover, the attentional, executive, language, and other academic deficits characteristic of NF1 may be reliant either in whole or in part on frontal lobe functions.

By using multiple DTI measures, this study uniquely contributes to our broader understanding of the previously observed white matter volume increases. Our finding of increased ADC reflects a general increase in diffusivity that can arise through a variety of mechanisms (see Figure 6). One post-mortem study using ADC alone has proposed that the increase in ADC commonly reported in NF1 could be result of increased vacuoles in the myelin [56]. This explanation has been used to explain subsequent findings of ADC increases [57], as has the hypothesis that the ADC difference reflects demyelination [25], [27]. However, while ADC is sensitive to determining whether there is more global diffusion, it cannot inform whether diffusion in a particular direction is differentially impacted, as can be assessed using FA. Our finding of significantly decreased FA in patients with NF1 indicates that diffusion is not globally increased, but is specifically less constrained perpendicular to the tracts (the ellipse describing the diffusion is less eccentric, or narrow, than in controls). Because of the directionality of this effect, it is unlikely that an increase in diffusion within circular vacuoules, which would not be associated with diffusion changes in any particular direction, could explain the observed differences. However, the finding of decreased FA may still be driven by either decreased organization of tracts, which prevents diffusion along the long (axial) axis of the ellipse, or alternatively by decreased myelination or increased axonal spacing that allows for more room between axons for water molecules to move perpendicular to the tract (radially). Therefore, the separate investigation of radial and axial diffusivity measures was critical to help elucidate the basis of the white matter microstructural changes. We demonstrated that within the tracts showing significant FA alteration the change is driven by radial diffusion with no significant change in axial diffusion. Thus, we have evidence indicating that the cause of the differences in FA and ADC between groups is likely based on either myelination differences, density of axonal packing, or both. Further, additional analyses using radial and axial diffusion as covariates had different effects on the significance of the FA analysis, further indicating that radial diffusion may play a particularly important role.

A limitation of DTI methodology is that it is an indirect measure of the distance between axons, and must remain agnostic to which factor (myelin or axonal spacing) is driving the results. However, given that white matter volume is increased in this sample and in other samples in the literature, a simple finding of decreased myelination (which would typically result in a decrease in white matter volume) is not sufficient to explain the larger pattern of results. Therefore, our data support the novel hypothesis that while there may be myelination differences in NF1, there are also likely to be differences in the degree of axonal packing, with the larger distances between axons contributing to the white matter volume increase. Given our findings of increased ADC and radial diffusion in the context of decreased FA, this hypothesis is consistent with histological findings that axonal packing density (amount of space between axons) is positively correlated with ADC and radial diffusion and negatively correlated with FA [36], [37], [38].

The possibility that the alterations in axial diffusion may be the result of increased space between axons (less dense tract packing) is supported by some of the known functions of neurofibromin and the molecules it interacts with. Developmentally, when white matter tracts are formed, cellular adhesion molecules play an important role in axon guidance along tracts, and in holding tracts together. There is some evidence that OMgp has the potential to function as an adhesion molecule [58], [59], and thus may be affected by NF1 mutation. OMgp expression across development coincides with the progression of myelination, which moves in a caudal to rostral direction [59], and it may have a particularly important effect in thalamocortical tracts (such as the ATR found to be significantly affected in the current study) [60]. Furthermore, neurofibromin itself has been shown to regulate not only Ras, but also Akt and focal adhesion kinase (FAK), which impacts cell adhesion, migration, and survival [61], [62]. While the current data cannot directly address this issue, the findings are consistent with a disruption in fasciculation during early development, and future histological and molecular studies addressing the relationship of these factors to white matter alterations will be highly informative.

Certain limitations of the current study must be noted. First of all, the sample size is small, due to limitations in recruiting individuals with this rare disorder and future studies in larger populations, as well as prospective longitudinal studies in developmental samples, will be useful for further probing the etiology and developmental trajectory of structural brain changes in NF1. Secondly, our DTI acquisition sequence had a limited number of directions and thus, we were not able to do tractography analyses. Further, while an increasing number of studies are beginning to include measures of radial and axial diffusion, as they provide more specific information about the nature of white matter microstructural changes than do FA and ADC, there are limitations to these measures. One particular issue is related to the reliability of these measures in areas where there are crossing fibers. This issue may be intensified in low directional data, as we have here. While our results indicate widespread changes across large areas of white matter, regional interpretation of these measures should be considered with caution. This may be better addressed in future studies with higher directional data. Finally, particularly interesting for future studies will be explorations of the relationship of these changes to clinical symptoms, such as those associated with ADHD. Although no studies, to our knowledge, have previously examined the impact of ADHD diagnosis on DTI indices in NF1, Cutting et al. [12] noted that NF1 children with an ADHD diagnosis had smaller frontal gray matter volumes relative to NF1 patients without ADHD. Similarly, it is possible that those individuals with the greatest deficits in white matter organization are also those for whom NF1 has resulted in ADHD symptoms. Based on the literature in idiopathic ADHD showing white matter deficits in prefrontal regions [63], [64], [65], we would anticipate that alterations in these regions in NF1 may also be associated with attentional symptoms. However, unlike in NF1, such studies have revealed deficits in axial as well as radial diffusion, Thus, further longitudinal developmental studies aimed at elucidating the similarities and differences in frontal lobe white matter development in NF1 and ADHD are warranted.

Overall, the current study demonstrates that the finding of white matter overgrowth, as measured by sMRI, persists into adulthood in NF1 patients and that white matter microstructural changes, as measured by DTI, also persist and are more pronounced in the frontal lobe. Notably, we show a novel pattern of DTI findings that may indicate that one contributing factor to this increase in volume may be related to looser fiber packing. Further, our findings indicate that indices of axial and radial diffusivity have added utility as markers of NF1-associated brain pathology beyond the typically investigated measures of diffusion (i.e., fractional anisotropy and ADC).
